# Wrist tendon moment arms: Quantification by imaging and experimental techniques

**DOI:** 10.1016/j.jbiomech.2017.12.024

**Published:** 2018-02-08

**Authors:** Angela K. Garland, Darshan S. Shah, Angela E. Kedgley

**Affiliations:** Department of Bioengineering, Imperial College London, London, United Kingdom

**Keywords:** Wrist, Moment arms, Imaging, MRI, Tendon excursion

## Abstract

Subject-specific musculoskeletal models require accurate values of muscle moment arms. The aim of this study was to compare moment arms of wrist tendons obtained from non-invasive magnetic resonance imaging (MRI) to those obtained from an in vitro experimental approach. MRI was performed on ten upper limb cadaveric specimens to obtain the centrelines for the flexor carpi radialis (FCR), flexor carpi ulnaris (FCU), extensor carpi radialis longus (ECRL), extensor carpi radialis brevis (ECRB), extensor carpi ulnaris (ECU), and abductor pollicis longus (APL) tendons. From these, the anatomical moment arms about each of the flexion-extension (FE) and radioulnar deviation (RUD) axes of the wrist were calculated. Specimens were mounted on a physiologic wrist simulator to obtain functional measurements of the moment arms using the tendon excursion method. No differences were observed between anatomical and functional values of the FE and RUD moment arms of FCR, ECRL and ECRB, and the RUD moment arm of ECU (p > .075). Scaling the anatomical moment arms relative to ECRB in FE and ECU in RUD reduced differences in the FE moment arm of FCU and the RUD moment arm of APL to less than 15% (p > .139). However, differences persisted in moment arms of FCU in RUD, and ECU and APL in FE (p < .008). This study shows that while measurements of moment arms of wrist tendons using imaging do not always conform to values obtained using in vitro experimental approaches, a stricter protocol could result in the acquisition of subject-specific moment arms to personalise musculoskeletal models.

## 1 Introduction

The accurate measurement of tendon moment arms is important for the development of musculoskeletal models. While generic models may utilise values obtained from the literature, the recent rise in subject-specific modelling necessitates quantification on an individual basis. Imaging techniques, such as magnetic resonance imaging (MRI), could enable non-invasive estimation of such parameters. Previous studies have reported the estimation of tendon moment arms using imaging at the third metacarpophalangeal joint in the hand (Wilson et al., 1999), at the ankle (Fath et al., 2010, Maganaris et al., 2000, Rugg et al., 1990) and the knee (Arnold et al., 2000). Some studies have reported differences of over 30% between tendon moment arms obtained from imaging as compared to those calculated using the tendon excursion method (Fath et al., 2010, Fowler et al., 2001), an experimental technique (An et al., 1983) that cannot be performed in vivo. The accuracy of such measurements from imaging has not been determined for the wrist, possibly because the wrist has two degrees of rotation – flexion-extension (FE) and radioulnar deviation (RUD) – and hence, two moment arms for each tendon.

The aim of this study was to compare anatomical moment arms of wrist tendons obtained from MRI with functional moment arms obtained using the tendon excursion method to determine the potential for accurate measurement of subject-specific moment arms using imaging alone. The hypothesis was that there would be no differences between the anatomical moment arms, quantified from imaging, and their corresponding functional equivalents.

## 2 Materials and methods

Ten fresh-frozen cadaveric upper limb specimens (eight females and two males, aged 49.7 ± 10.4 years) were obtained from a licensed human tissue facility. Ethical approval for the use of these specimens was obtained from the Tissue Management Committee of the Imperial College Healthcare Tissue Bank, according to the Human Tissue Act.

### 2.1 Anatomical moment arm estimations using MRI

**Fig. 1:**
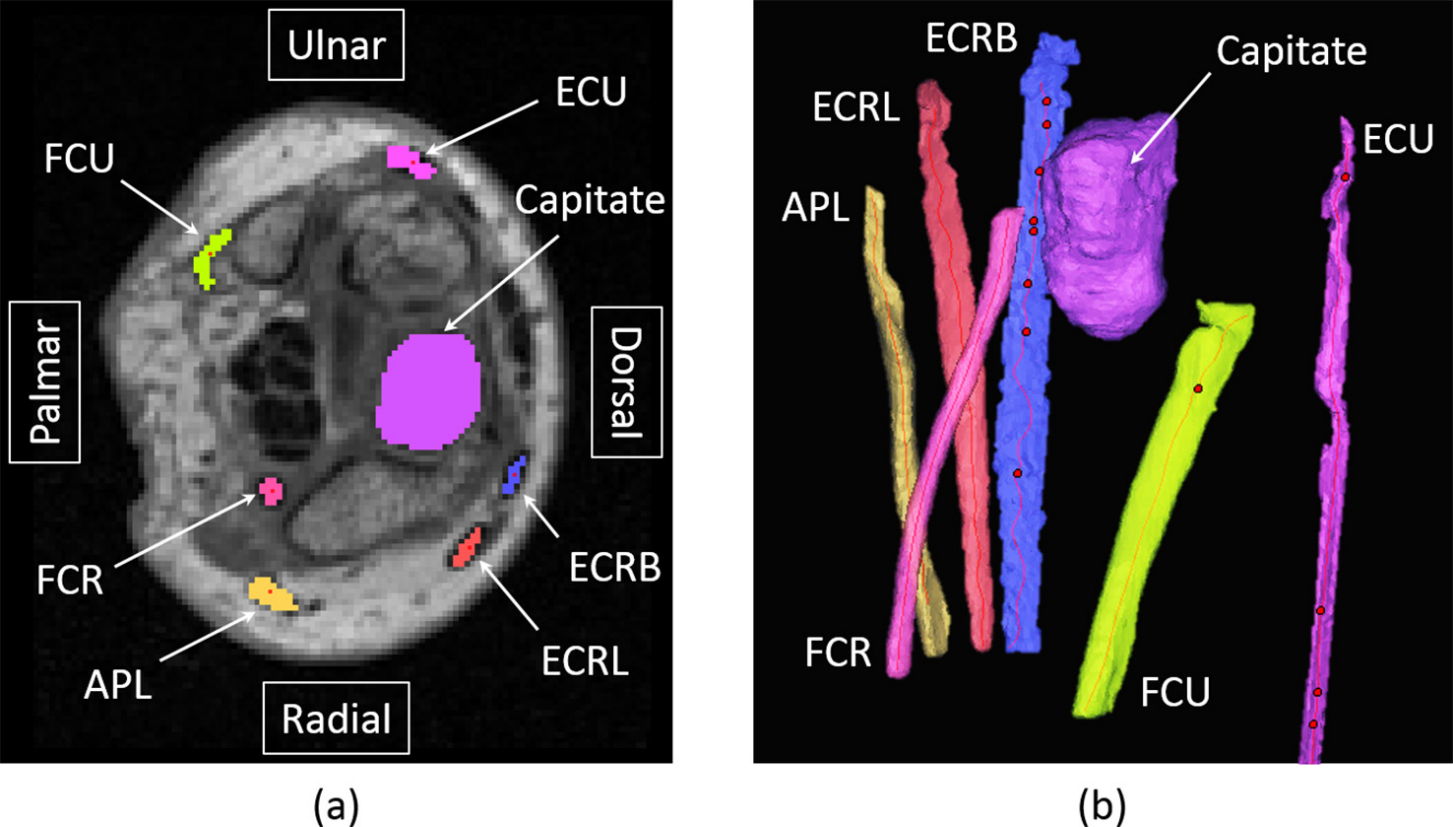
The capitate and tendons of the wrist, with centrelines, as seen in (a) a transverse section of a magnetic resonance imaging (MRI) scan and (b) 3D reconstructions from the MRI scan. Moment arms of the tendons of the flexor carpi radialis (FCR), flexor carpi ulnaris (FCU), extensor carpi radialis longus (ECRL), extensor carpi radialis brevis (ECRB), extensor carpi ulnaris (ECU), abductor pollicis longus (APL) were quantified.

MRI scans (MAGNETOM Aera, 1.5T, Siemens AG, Munich, Germany; DIXON sequence, TR = 7.36 ms, TE = 2.39 ms, coronal plane resolution of 0.65 mm, slice thickness of 0.64 mm) of the specimens, with the forearm in a supinated position, were obtained. Tendons and bony anatomy were segmented (Mimics Research, Materialise, Leuven, Belgium) (Fig. 1). To replicate their lines of action, centrelines were created for flexor carpi radialis (FCR), flexor carpi ulnaris (FCU), extensor carpi radialis longus (ECRL), extensor carpi radialis brevis (ECRB), extensor carpi ulnaris (ECU), and abductor pollicis longus (APL) tendons. Vectors describing the tendon paths were defined using two points on the centrelines – the distal point was taken at the level of the proximal head of the capitate in the transverse plane (Youm et al., 1978), while the proximal point was defined 30 mm from the distal point along the tendon centreline.

Co-ordinate frames for the hand and forearm were created from anatomical bony landmarks (Wu et al., 2005). The co-ordinate frame for the hand was rotated to achieve the neutral wrist position (FE = 0°, RUD = 0°), as per Garner and Pandy (1999). The concave curvature of the distal radius provided the position and orientation of the FE axis (Garner and Pandy, 1999). The RUD axis was defined by the cross product of the FE axis and the long axis of the third metacarpal, followed by a 5 mm distal displacement in the direction of the long axis of the third metacarpal (Andrews and Youm, 1979, Charles and Hogan, 2011, Garner and Pandy, 1999, Youm and Yoon, 1979). The FE and RUD moment arms of each tendon were calculated as the minimum orthogonal distance between the tendon centrelines and the respective axes, using a custom-written MATLAB (Mathworks, Natick, USA) code.

### 2.2 Functional moment arm estimations using the tendon excursion method

The specimens, stored at −20 °C prior to testing, were thawed at room temperature for 12 h. The six wrist muscles considered for this study – FCR, FCU, ECRL, ECRB, ECU and APL – were dissected at their distal musculotendinous junction while all soft tissue was resected 5 cm proximal to the wrist. The specimens were then mounted onto a physiologic wrist simulator (Shah and Kedgley, 2016, Shah et al., 2017) with the distal tendons of the wrist muscles connected to electromechanical actuators (SMS Machine Automation, Barnsley, UK) via steel cables. The wrist was passively moved through two cycles of FE or RUD, with the end points for the limits of the range of motion imposed by the specimen. A custom-written LabVIEW (National Instruments Corp., Austin, USA) code prevented the tendons from unloading. An eight-camera optical motion tracking system (Qualisys, Göteborg, Sweden) was used to quantify wrist kinematics and displacement of each actuator was used to quantify tendon excursion. FE and RUD moment arms of each tendon were calculated according to the tendon excursion method (An et al., 1983). Mean values of tendon moment arms across the range of motion were compared to those found using MRI, and those reported in the literature (Brand and Hollister, 1999, Horii et al., 1993, Loren et al., 1996).

### 2.3 Scaling moment arms obtained from MRI

A scaling approach was trialled to improve the accuracy of the moment arms obtained from MRI. The tendon with the smallest difference between anatomical and functional moment arms in each of FE and RUD was selected as the ‘base’ tendon. Scaling factors were calculated by taking the ratio of the anatomical moment arms of each tendon with respect to the corresponding base tendon in FE and RUD. Specimens for which the scaling ratio differed by more than 30% from the mean were designated as outliers and were excluded. Thus, a maximum of two outliers were neglected for each tendon, before calculating the mean scaling ratios across all specimens for each tendon. Scaled moment arms were then calculated for each tendon for each specimen by multiplying the anatomical moment arm obtained from imaging to the corresponding scaling factor.

### 2.4 Statistical analysis

The data were analysed using the Shapiro-Wilk test for normality and compared using one-way repeated measures analyses of variance (ANOVA) (IBM SPSS Statistics, IBM Corp., Armonk, USA). Significance was defined as p < .05.

## 3 Results

The maximum inter-subject variability, quantified by the standard deviation (SD) of moment arms, was observed for the RUD moment arm of FCU using both the tendon excursion method (SD = 4.2 mm) and MRI (SD = 6.6 mm) (Table 1).

**Table 1:** Comparison of the measured and scaled values of flexion-extension (FE) and radioulnar deviation (RUD) moment arms obtained using magnetic resonance imaging (MRI) with the tendon excursion (TE) method for the flexor carpi radialis (FCR), flexor carpi ulnaris (FCU), extensor carpi radialis longus (ECRL), extensor carpi radialis brevis (ECRB), extensor carpi ulnaris (ECU), and abductor pollicis longus (APL) for 10 specimens. FE moment arms were scaled with respect to the ECRB and RUD moment arms were scaled with respect to the ECU. Data are represented as mean ± standard deviation. Flexion is positive and extension is negative in FE. Ulnar deviation is positive and radial deviation is negative in RUD. Significance was defined as p < .05.

	Technique	Tendon					
		FCR	FCU	ECRL	ECRB	ECU	APL
FE moment arm (mm)	TE	12.2 ± 2.1	11.3 ± 2.1	−6.6 ± 3.0	−10.8 ± 2.8	−4.0 ± 0.9	6.8 ± 1.9
	MRI measured	16.2 ± 1.5	14.8 ± 4.3	−4.8 ± 1.3	−9.2 ± 1.6	−10.2 ± 3.2	10.7 ± 4.0
	p-value	0.097	0.021	0.075	0.227	0.001	0.013
	Scaling ratio	−1.80	−1.42	−0.47	–	1.01	−1.06
	MRI scaled	16.6 ± 2.8	13.1 ± 2.2	−4.3 ± 0.7	–	−9.3 ± 1.6	9.8 ± 1.7
	p-value	0.013	0.139	0.114	–	0.005	0.019

RUD moment arm (mm)	TE	−10.2 ± 2.5	11.7 ± 4.2	−17.2 ± 3.6	−10.0 ± 2.2	23.7 ± 3.3	−23.6 ± 2.5
	MRI measured	−8.6 ± 4.0	19.8 ± 6.6	−16.7 ± 2.4	−11.1 ± 2.6	23.0 ± 3.1	−19.7 ± 3.9
	p-value	0.226	0.01	0.687	0.156	0.484	0.023
	Scaling ratio	−0.30	0.72	−0.80	−0.44	–	−0.98
	MRI scaled	−6.8 ± 0.9	16.6 ± 2.2	−18.3 ± 2.4	−10.0 ± 1.3	–	−22.6 ± 3.0
	p-value	0.003	0.004	0.380	0.943	–	0.223

No differences were observed between the anatomical and functional moment arms for FCR, ECRL, ECRB, and the RUD component of ECU (p > .075) (Table 1). However, functional moment arms differed from the anatomical values for FCU in FE (23.8%) and RUD (41.1%), ECU in FE (60.9%), and APL in FE (36.5%) and RUD (16.3%) (p < .023).

ECRB and ECU were selected as ‘base’ tendons for scaling in FE and RUD, respectively (Table 1). Scaling reduced the differences between anatomical and functional moment arms for FCU in FE (14.2%) and RUD (29.7%), ECU in FE (57.2%), and APL in FE (30.3%) and RUD (10.9%). Despite this, differences persisted for FCU in RUD, and ECU and APL in FE (p < .008).

## 4 Discussion

**Fig. 2:**
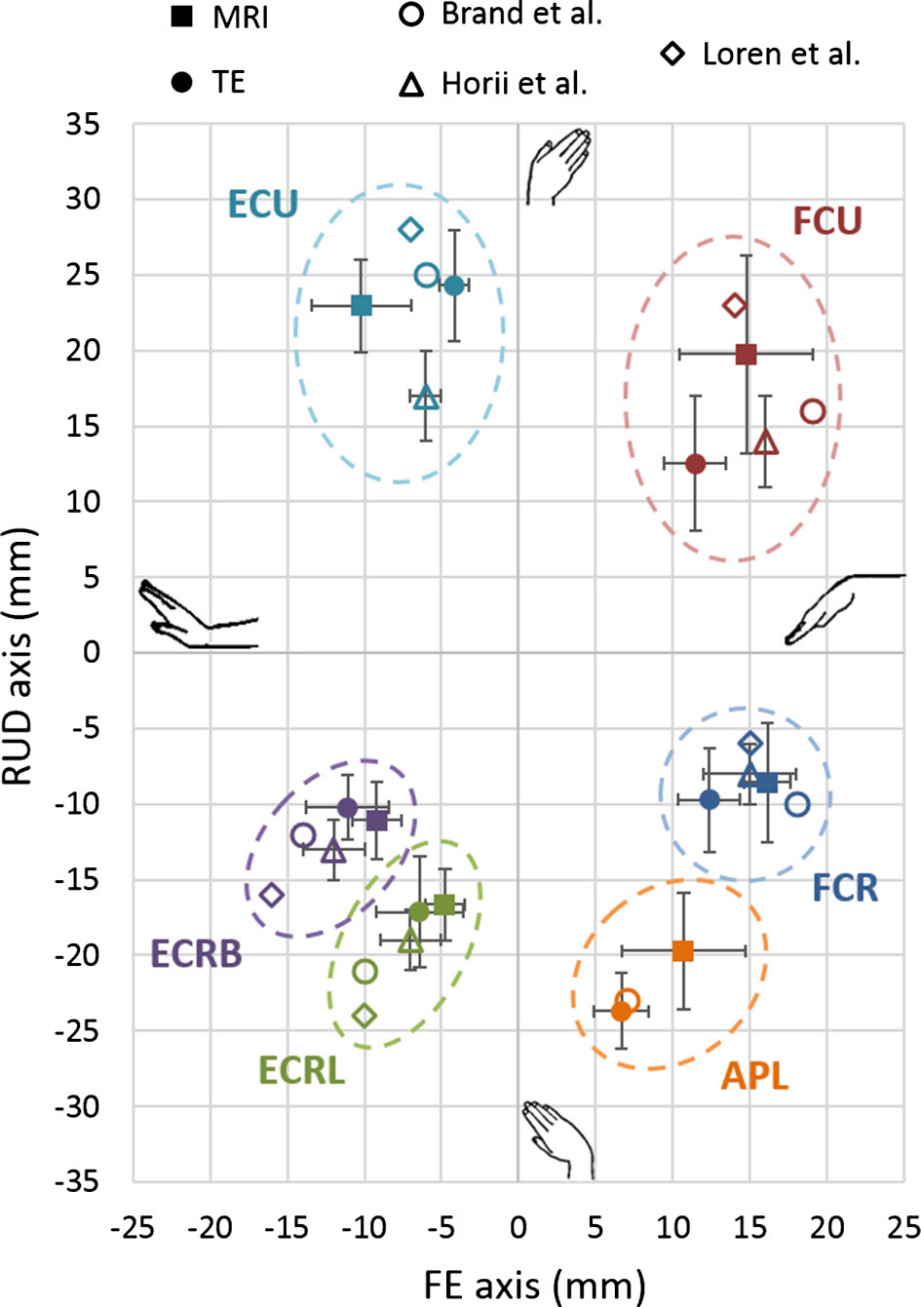
Comparison of moment arms in flexion extension (FE) and radioulnar deviation (RUD) obtained using magnetic resonance imaging (MRI) and the tendon excursion method (TE) for 10 specimens with data from Brand and Hollister (1993) for 11 specimens, Horii et al. (1993) for 7 specimens and Loren et al. (1996) for 5 specimens for the flexor carpi radialis (FCR), flexor carpi ulnaris (FCU), extensor carpi radialis longus (ECRL), extensor carpi radialis brevis (ECRB), extensor carpi ulnaris (ECU), abductor pollicis longus (APL).

This study compared the anatomical moment arms of six wrist tendons obtained using MRI to the functional moment arms calculated using the tendon excursion method. Since these tendons have relatively constant moment arms (Brand and Hollister, 1999, Horii et al., 1993), mean values of functional moment arms across the ranges of motion were used to compare the corresponding anatomical moment arms. Functional moment arms were within two standard deviations of those reported in other experimental studies on the wrist (Brand and Hollister, 1999, Horii et al., 1993, Loren et al., 1996) (Fig. 2). Despite the complexity associated with having two degrees of rotation in the wrist, which gives rise to two moment arms for each tendon, no differences were observed between anatomical and functional moment arms of FCR, ECRL and ECRB (p > .075).

Differences between anatomical and functional moment arms could arise for a number of reasons. Errors in the segmentation of bony landmarks and tendon paths from MRI influence the calculations of both joint axes and moment arms (Wilson et al., 1999). In order to avoid the local divergence of FCR, ECRL, ECRB, ECU and APL close to their insertions on the metacarpals, tendon vector distal points were selected in the transverse plane passing through the capitate head. However, in the case of FCU, to minimise the differences with respect to the functional moment arms, the tendon vector distal point was chosen 10 mm proximal to the capitate head, owing to its insertion on the pisiform. Since the selection of the proximal point of the tendon vector did not greatly affect the moment arms of the wrist tendons (Appendix A.1), the proximal point of all tendon vectors was chosen at 30 mm from the distal point, in order to best characterise the paths of the wrist tendons. The effect of the inclusion of soft tissue on the quantification of anatomical moment arms was also insignificant (Appendix A.2).

Differences between the anatomical and functional FE and RUD moment arms of FCU and APL, and the FE moment arm of the ECU (p < .023) were likely due to the inherent tendon anatomy. Since FCU inserts on the pisiform, the tensile load applied to the tendon could affect the quantification of the moment arm (Olszewski et al., 2015). Actuators were used to apply tensile loads to FCU, which were similar to those applied by dead weights in the literature (Brand and Hollister, 1999, Horii et al., 1993, Loren et al., 1996); however, anatomical moment arms obtained from MRI were quantified without contraction of muscle. In the case of APL, the discrepancy between anatomical and functional moment arms could be attributed to the numerous slips of the distal tendon (Thwin et al., 2014). Although careful specimen dissection proximal to the retinaculum ensured consideration of all slips experimentally, the presence of several slips could make it difficult to select and accurately distinguish all slips of the distal APL tendon using MRI. Lastly, the FE moment arm of the ECU varies with the degree of forearm pronation, with the moment arm in supination being more than twice that in pronation (Brand, 1974, Horii et al., 1993). Since the forearm was placed in the mid-prone position (pronation = 0°) for the tendon excursion test, the functional FE moment arm of ECU agrees well with other experimental studies (Brand and Hollister, 1999, Horii et al., 1993, Loren et al., 1996) (Fig. 2). However, it is lower than the anatomical FE moment arm of ECU (p = .001) obtained from the MRI of a supinated forearm, which is closer to values reported for functional moment arms for a supinated forearm (Brand, 1974, Horii et al., 1993).

ECRB and ECU were used as ‘base’ tendons in FE and RUD, respectively, since there were no differences between their anatomical and functional moment arms (p > .227) (Table 1). When the anatomical moment arms of the remaining tendons were scaled with respect to the base tendons, differences between the anatomical and functional values in both FE and RUD for FCU and APL were reduced. Subsequently, there were no differences in the FE moment arm of FCU and the RUD moment arm of APL (p > .079). Differences between the anatomical and functional moment arms for FCU in RUD and APL in FE, although significant (p < .019), were less than 30%, which was similar to comparative studies performed on other joints (Arnold et al., 2000, Fath et al., 2010, Fowler et al., 2001, Wilson et al., 1999). Scaling increased the difference between the anatomical and functional values of FCR and ECRL. Thus, in order to improve moment arm measurements of wrist tendons from MRI, scaling should be used only for FCU and APL.

A shift in the position and orientation of the FE and RUD axes would affect the corresponding moment arms; hence, the use of a generic definition of the anatomical axes in this study could be another cause of the discrepancies between anatomical and functional moment arms. The use of subject-specific helical axes (Salvia et al., 2000, Sommer and Miller, 1980, Woltring et al., 1985) could provide more accurate estimations of anatomical moment arms. Moreover, FE and RUD moment arms of wrist tendons have been used to replicate the complex functional wrist motions, such as the dart thrower’s motion (DTM) and circumduction, in vitro, as a combination of FE and RUD (Shah et al., 2017); however, since DTM has been reported to be a planar motion (Moritomo et al., 2014), subject-specific anatomical moment arms could also be calculated about the functional DTM axis in vivo, in the future.

In summary, for both components of FCR, ECRL, ECRB, and the RUD component of ECU, anatomical moment arms measured from MRI did not differ from functional moment arms obtained using the tendon excursion method. Thus, these moment arms could be used directly in the customisation of musculoskeletal models. For FCU and APL, scaling the anatomical moment arms improved their conformance to the functional moment arms; however, differences persisted. Therefore, while the scaling ratios presented herein may be used to estimate subject-specific tendon moment arms, it should be noted that variations of up to 30% may be present. Owing to the dependence on the forearm pronation angle in the case of the FE moment arm of the ECU, and the presence of multiple tendon slips in the case of APL, it is vital to decide these parameters before quantifying the moment arms of these tendons in vitro. The results of this study show that the moment arms of wrist tendons, which substantially influence the output of musculoskeletal models, may be quantified in vivo; however, these might differ from the corresponding functional moment arm values. Therefore, for applications requiring precise customisation of musculoskeletal models, it is recommended to implement stricter imaging protocols, as well as define specimen-specific helical axes, which could avoid discrepancies between the anatomical moment arms measured from imaging and the functional moment arms obtained in vitro.
