# Supplementary material for: Wrist tendon moment arms: Quantification by imaging and experimental techniques
Source: J Biomech. 2018 Feb 8;68:136–40. doi: 10.1016/j.jbiomech.2017.12.024 (PMC5793998; doi:10.1016/j.jbiomech.2017.12.024)
Supplement: Supplementary data 1 [file mmc1.docx]

# Wrist tendon moment arms: Quantification by imaging and experimental techniques

Angela K Garland, Darshan S Shah, Angela E Kedgley

## Appendix A: Sensitivity of measurements in MRI

### A.1 – Selection of end points for tendon paths

Following the definitions of vectors describing the tendon paths along the centrelines of the tendons (section 2.1), a sensitivity analysis was carried out to investigate the effect of the chosen end points of the tendon vectors on the resultant moment arms. For all six tendons, the vectors were calculated with the proximal point at 10mm, 20mm and 30mm from the distal point, while keeping the distal point constant. In case of the FCU, an additional analysis was performed, whereby the distal point was moved proximally by 5mm, 10mm, 15mm and 20mm for each of the proximal points described above.

Variations in flexion-extension (FE) and radioulnar deviation (RUD) moment arms due to the selection of the tendon vector proximal point were less than 10%, except for the FE moment arm of ECRL (Table A1). Therefore, the proximal point of all tendon vectors was chosen at 30mm from the distal point on the tendon centreline. The distal point of the FCU tendon vector was selected at 10mm proximal to the transverse plane passing through the head of the capitate, since this minimised the difference between the anatomical and functional moment arms (Table A2).

Table A1: Maximum change in the flexion-extension (FE) and radioulnar deviation (RUD) moment arms obtained by moving the proximal point of the tendon vectors by 10mm, 20mm, and 30mm for the flexor carpi radialis (FCR), extensor carpi radialis longus (ECRL), extensor carpi radialis brevis (ECRB), extensor carpi ulnaris (ECU), abductor pollicis longus (APL) with their distal point fixed at the level of the head of the capitate in the transverse plane. Data are represented as mean ± standard deviation (percentage change) for 10 specimens.

|  | **FCR** | **ECRL** | **ECRB** | **ECU** | **APL** |
| --- | --- | --- | --- | --- | --- |
| **Change in FE moment arm (mm)** | 0.37 ± 0.47 (2.3%) | 0.75 ± 0.69 (15.7%) | 0.35 ± 0.27 (3.8%) | 0.93 ± 0.83 (9.1%) | 0.85 ± 0.95 (7.9%) |
| **Change in RUD moment arm (mm)** | 0.19 ± 0.23 (2.2%) | 1.03 ± 1.37 (6.2%) | 0.24 ± 0.24 (2.2%) | 0.73 ± 0.91 (3.2%) | 1.25 ± 1.67 (6.3%) |

Table A2: Maximum change in the flexion-extension (FE) and radioulnar deviation (RUD) moment arms obtained by moving the proximal point of the tendon vectors by 10mm, 20mm, and 30mm for the flexor carpi ulnaris (FCU) with the distal point moved in 5mm increments proximally from the level of the head of the capitate in the transverse plane. Data are represented as mean ± standard deviation (percentage change) for 10 specimens.

|  | **Proximal shift of distal point of FCU tendon vector (mm)** | | | | |
| --- | --- | --- | --- | --- | --- |
|  | **0** | **5** | **10** | **15** | **20** |
| **Change in FE moment arm (mm)** | 0.37 ± 0.39 (2.5%) | 0.11 ± 0.10  (0.8%) | 0.87 ± 0.95 (5.9%) | 0.37 ± 0.40 (2.5%) | 0.42 ± 0.41 (2.6%) |
| **Change in RUD moment arm (mm)** | 1.00 ± 0.63 (5.1%) | 0.41 ± 0.44  (2.1%) | 0.65 ± 1.19 (3.3%) | 0.87 ± 0.84 (4.5%) | 1.13 ± 0.92 (5.7%) |

### A.2 – Effect of soft tissue on co-ordinate frames of reference

Anatomical bony landmarks used to create co-ordinate frames for the hand and forearm (section 2.1) were digitised on the surfaces of the bones in the MRI. In the case of the tendon excursion method, however, these landmarks were located on the skin. Hence, to determine the effect of the layers of soft tissue on the moment arm calculations, the co-ordinate frames used for the tendon excursion test were replicated on the MRI scans by selecting points on the skin above the locations of the bony landmarks.

This change in the co-ordinate frame of the hand did not affect the FE axis, which was calculated using the curvature of the radius (Garner & Pandy, 1999). Hence, no changes were observed in FE moment arms. Although the change in the co-ordinate frame of the hand affected the calculated RUD axis, the maximum change observed in moment arms of the tendons was 0.1 mm (1.2%). Thus, use of markers placed on the skin did not greatly affect the moment arms of the tendons. Consequently, we hypothesize that the effect of inclusion or compression of soft tissue would have no effects on the moment arm calculations.
